# Tackling Publication Bias and Selective Reporting in Health Informatics Research: Register your eHealth Trials in the International eHealth Studies Registry

**DOI:** 10.2196/jmir.6.3.e35

**Published:** 2004-09-30

**Authors:** Gunther Eysenbach

**Affiliations:** ^1^Centre for Global eHealth InnovationTorontoCanada

**Keywords:** Clinical protocols, publication bias, randomized controlled trials/standards, registries, information storage and retrieval

## Abstract

Beginning in July 2005, several major medical journals, including the Journal of Medical Internet Research, will only consider trials for publication that have been registered in a trial registry before they started. This is to reduce publication bias and to prevent selective reporting of positive outcomes. As existing clinical trial registers seem to be unsuitable or suboptimal for eHealth studies, a free International eHealth Study Registry (IESR) has been set up, allowing registration of trials (including non-randomized studies) in the field of health informatics and assigning an International eHealth Study Number (IESN). The IESR should meet the requirements of journal editors for a-priori registration of a study. We hope IESR will become the preferred choice for registration of eHealth studies and, as an secondary benefit, will become an international repository of ongoing eHealth projects, thereby enhancing global collaboration and reducing duplication of effort.

## Compulsory Registration of Studies as Requirement for Publication

This month some of the world's leading medical journals under the umbrella of the International Committee of Medical Journal Editors (ICMJE) made an important and far-reaching announcement: they will not publish trials in the future unless they have been registered before they started [1]. To be considered for publication in these journals, trials that begin enrollment of patients after July 1, 2005 must register in a trials registry at or before the onset of enrollment. For trials that begin enrolment before this date, the journals will require registration by September 13, 2005. (See multimedia appendix for the ICMJE statement). With this measure, editors of journals hope to effect widespread registration of trials to counter selective reporting and publication bias. JMIR will join this initiative and will (after July 1, 2005) publish only randomized trials or cohort studies which have been registered before starting enrollment. JMIR has also created a registry for eHealth studies and urges all eHealth researchers to register their planned or ongoing projects, regardless of whether they intend to submit them to this or other journals.

The mandatory registration of clinical trials has been demanded for almost 20 years and is overdue [2-4]. The new requirement from many of the world's leading journals is a breakthrough for ensuring the quality of clinical research. It is long known that negative trials are less likely to be published than positive trials [4], and that this leads to a problem called “publication bias”, with somebody just appraising the published literature coming to a more positive conclusion about the effectiveness of an intervention than somebody who would be aware of all trial results. Although widespread use of trial registers will not prevent negative trials from remaining unpublished, it will at least help systematic reviewers identify unpublished trials and will improve the quality of published study reports. While the Internet has already been a very useful tool helping systematic reviewers discover ongoing and planned research, this has required tedious “detective work” for systematic reviewers to find clues on the homepages of researchers and funding agencies [5]. With Web-based trial registers, investigators will now leave digital trails on the Internet so that knowledge synthesis researchers can contact the investigators for further information. Furthermore – and perhaps even more importantly – registration of key data such as the primary outcome measures and trial duration before the trial starts may prevent post hoc “data dredging” (fishing for significance) or selective reporting.

A recent high-profile case of alleged selective reporting was the drug company- sponsored CLASS trial, which compared gastrointestinal toxicity of Celecoxib against other nonsteroidal anti-inflammatory drugs (NSAIDs) [6]. Investigators were accused of intentionally misleading readers by reporting only the more favourable 6-month outcomes for a trial that lasted 12 months – a fact that was not reported in the final publication [7,8]. According to critics of the publication, most of the ulcer complications occurred in the second half of the study period, and if 12-month outcome data had been compared, some of the drug's apparent safety advantage would have been diminished. The investigators deny any wrongdoing and said that the reported data “best reflected the comparisons they were trying to make [9].”

The prevalence of cases where pharmaceutical companies try to intentionally mislead peer reviewers and the public is unknown. However, investigator-driven, well-intentioned selective reporting is likely to be widespread. In an attempt to make their manuscripts more interesting and to increase their chances of acceptance by journals, investigators almost routinely highlight the positive findings and sometimes do not mention the negative outcomes. Not reporting all the negative findings is, of course, is a problem, as it conceals the fact that the positive result could be spurious finding: If investigators make 20 different comparisons (eg, measure 5 outcomes on 4 different points in time) at least one will be statistically significant on a 5% level by chance alone. If investigators report only this one “positive” comparison, without mentioning that they made 19 other comparisons which were all negative, the reader is misled. In one recent analysis, where the protocols of studies submitted to an institutional review board were systematically compared against publications of these studies, 62% of at least primary outcome was changed, introduced, or omitted. On average 50% of efficacy and 65% of harm outcomes per trial were incompletely reported; and statistically significant outcomes were more likely to be reported than non-significant results [10]. In health informatics, where researchers often do exploratory studies by measuring multiple outcomes, selective reporting is likely to be highly prevalent.

## The Case for an eHealth Study Registry

Although the ICMJE initiative is exciting and is to be welcomed, those primarily interested in the evaluation of non-drug interventions (such as eHealth interventions) are left confused. The signatories of the ICMJE editorial define a clinical trial as “any research project that prospectively assigns human subjects to intervention or comparison groups to study the cause-and-effect relationship between a medical intervention and a health outcome.” Despite this seemingly broad definition, the ICMJE initiative and the surrounding discussion are focused on drug trials. This focus is demonstrated by the ICMJE's endorsement of clinicaltrials.gov as the preferred registry [11,12]. As others have pointed out [13], clinicaltrials.gov offers registration only to "(US) federal agencies sponsoring the clinical research studies (both interventional and observational trials), private sponsors that have submitted an Investigational New Drug Application (IND) to the Food and Drug Administration (FDA), such as pharmaceutical companies, and organizations representingIND sponsors.” Not only is the clinicaltrials.gov registry restricted to US-funded trials, it practically excludes most eHealth and health informatics trials, if they do not study regulated interventions such as drugs or medical devices.

Secondly, eHealth and medical informatics studies often look at more effective services, health services utilization or other variables related to the health care system as endpoints. It is uncertain whether such studies are covered by the definition of the ICMJE which focuses on “health outcomes”. To be on the safe side, and also to ensure eligibility for publication in JMIR, the British Medical Journal (BMJ) or other journals, we recommend that all researchers prospectively register their studies in a registry – but which one?

Although there are commercial trial registers available which provide alternatives to clinicaltrials.gov, these are not always the best choice for eHealth trials. One register, Current Controlled Trials (CCT), assigns, for a fee of about $150, an International Standard Randomized Controlled Trial Number (ISRCTN). This trial register does not meet the ICMJE requirements because it is private and for-profit and lacks backing by a public institution such as a university. Also, it does not meet some eHealth research community requirements, such as a health informatics-specific thesaurus to index the trials. Furthermore, the scope of CCT is “a clinical study in which two (or more) forms of care are compared, and in which the participants are allocated to one of the forms of care in the study, in an unbiased way, by using the play of chance.” Thus, this register focuses on “clinical care” (does this include home care?) and is restricted to randomized studies, while we think that other types of studies, which may be equally or more suitable in our field, should also be registered [14].

### The International eHealth Study Registry (IESR)

To meet the requirements of the eHealth and medical informatics community, we have set up an eHealth study registry on the JMIR site, which should meet the requirements of most journals. Our non-profit International eHealth Study Registry (IESR) will assign a International eHealth Study Number (IESN) to each submitted study. We hope IESR will become the preferred choice for registration of eHealth studies and, as an secondary benefit, will become an international repository of ongoing eHealth projects, thereby enhancing global collaboration and reducing duplication of effort.

Does it make sense to create yet another registry? Yes, because it is unlikely that only a single endorsed trial register will serve for all trials in the world. It is more likely (and this is partly a current reality) that multiple domain-, funder- or country-specific registers will exist. All will be accessible on the Internet and made interoperable and cross-searchable forming a large “Meta-Register”. In the end it will not matter where a trial or research project has been physically registered. This is similar to the Santa Fe Open Archives standards in Open Access publishing that enable harvesters to search across different archives. With this in mind, it seems important to add the criterion “interoperability” to the list of trial register requirements, which is neither mentioned by the ICMJE [1] nor the BMJ [13].

In addition to developing an eHealth study-specific thesaurus and indexing system based on registry submissions, other innovations distinguish IESR from generic registries such as clinicaltrials.gov or CCT. For example, we will provide a “results” field in the database, making it easy for registrants of the eHealth research to report their results in a very short form or to link to subsequent publications. In addition, the register will have a one-click “submit for publication” button to submit the protocol with the short results for publication to JMIR. The report will then be peer reviewed and can be published as a short report or letter to the editor, so that it can be indexed in bibliographic databases such as Medline. The rationale for this feature is that health informatics is an area in which a significant proportion of research regarding, for example, introduction of information technology in hospitals or provision of eHealth gadgets to consumers remains unpublished [11]. In many cases authors never write up research because of lack of time or motivation, and this “one-click-submit-for-publication” feature may encourage authors to publish their findings at least as a short report.

### Scope of the IESR

It is important to understand that the scope of the registry is wider than registration of eHealth studies intended for publication in JMIR. We hope that the registry becomes a database of planned and ongoing research where all studies related to information and communication technologies (ICT) in health are submitted, regardless of where authors plan to submit the results for publication. We define an eHealth study as any type of empirical research, evaluation and development activity studying the effect of ICT interventions in a health or health services context. ICT includes Internet and Intranet applications, studies of Web-based interventions, telehealth, telemedicine, clinical informatics applications (Hospital Information Systems, decision support) and consumer health informatics. Apart from RCTs we expect also other types of longitudinal studies or even cross-sectional and qualitative studies to be submitted and registered.

We recommend registering only concrete projects (ie, those which have already secured funding or are about to be started) as opposed to mere ideas. As described in a separate editorial [16], we are also offering peer review and publication of complete protocols in JMIR, but registration of the study in the IESR and publication of the protocol are separate processes and take place independently of each other.

### Registration Process

The registry (which is non-profit and hosted at the Centre for Global eHealth Innovation in Toronto, Canada) is a database which allows investigators (or their proxies, such as research associates or funding agencies) to publish their research protocol in an abbreviated format. The content will be reviewed by a registry editor, and the principal investigator [PI]) will be contacted to confirm the details of the study. Entries will not be copyedited or peer reviewed. The primary purpose is to disclose the important information from the protocol such as study question and endpoints to be measured prior to starting the trial. However, investigators can also add other information such as the profile of a desired collaborator.

The system will assign an unique International eHealth Study Number (IESR). The registry meets the criteria of journals such as JMIR or the British Medical Journal (BMJ). As clinicaltrials.gov does not accept non-US funded trials, it is anticipated that the signatories of the ICMJE editorial will also accept studies with protocols published in the IESR to meet their requirement of advance registration before a study can be published.

Investigators will be able (and will be encouraged) to continuously update their entries, with older versions being kept on file and retrievable for archival purposes.

The IESR database is designed to be complementary to other registries such as clinicaltrials.gov, not competitive. IESN will primarily contain studies that are not eligible for registration on clinicaltrials.gov. There will also be cross-links if a trial is also entered in other trial registers.

## The Goal: “Openness” and Excellence in eHealth Research

A natural synergy exists between an Open Access eHealth journal and a trial registry; both journal and registry share the common vision of enhancing access to research information and promoting “openness” of research processes and results. In combination with JMIR's new feature of offering peer review of research protocols [16], we hope that these will be important steps in our quest to improve the quality of eHealth research and to generate and disseminate high-quality evidence in the field.
