# Supplementary material for: Tackling Publication Bias and Selective Reporting in Health Informatics Research: Register your eHealth Trials in the International eHealth Studies Registry
Source: J Med Internet Res. 2004 Sep 30;6(3):e35. doi: 10.2196/jmir.6.3.e35 (PMC1550610; doi:10.2196/jmir.6.3.e35)
Supplement: Supplementary file 1 [file jmir_v6i3e35_app1.pdf]

Clinical Trial Registration: A Statement from the International Committee of Medical Journal Editors

Altruism and trust lie at the heart of research on human subjects. Altruistic individuals volunteer for research because they trust that their participation will contribute to improved health for others and that researchers will minimize risks to participants. In return for the altruism and trust that make clinical research possible, the research enterprise has an obligation to conduct research ethically and to report it honestly. Honest reporting begins with revealing the existence of all clinical studies, even those that reflect unfavorably on a research sponsor's product.

Unfortunately, selective reporting of trials does occur, and it distorts the body of evidence available for clinical decision making. Researchers (and journal editors) are generally most enthusiastic about the publication of trials that show either a large effect of a new treatment (positive trials) or equivalence of two approaches to treatment (noninferiority trials). Researchers (and journals) typically are less excited about trials that show that a new treatment is inferior to standard treatment (negative trials) and even less interested in trials that are neither clearly positive nor clearly negative, since inconclusive trials will not in themselves change practice. Irrespective of their scientific interest, trial results that place financial interests at risk are particularly likely to remain unpublished and hidden from public view. The interests of the sponsor or authors notwithstanding, anyone should be able to learn of any trial's existence and its important characteristics.

The case against selective reporting is particularly compelling for research that tests interventions that could enter mainstream clinical practice. Rather than a single trial, it is usually a body of evidence, consisting of many studies, that changes medical practice. When research sponsors or investigators conceal the presence of selected trials, these studies cannot influence the thinking of patients, clinicians, other researchers, and experts who write practice guidelines or decide on insurance-coverage policy. If all trials are registered in a public repository at their inception, every trial's existence is part of the public record and the many stakeholders in clinical research can explore the full range of clinical evidence. We are far from this ideal at present, since trial registration is largely voluntary, registry data sets and public access to them vary, and registries contain only a small proportion of trials. In this editorial, published simultaneously in all member journals, the International Committee of Medical Journal Editors (ICMJE) proposes comprehensive trials registration as a solution to the problem of selective awareness and announces that all 11 ICMJE member journals will adopt a trials-registration policy to promote this goal.

The ICMJE member journals will require, as a condition of consideration for publication, registration in a public trials registry. Trials must register at or before the onset of patient enrollment. This policy applies to any clinical trial starting enrollment after July 1, 2005. For trials that began enrollment prior to this date, the ICMJE member journals will require registration by September 13, 2005, before considering the trial for publication. We speak only for ourselves, but we encourage editors of other biomedical journals to adopt similar policies. For this purpose, the ICMJE defines a clinical trial as any research project that prospectively assigns human subjects to intervention or comparison groups to study the cause-and-effect relationship between a medical intervention and a health outcome. Studies designed for other purposes, such as to study pharmacokinetics or major toxicity (for example, phase I trials), would be exempt.

The ICMJE does not advocate one particular registry, but its member journals will require authors to register their trial in a registry that meets several criteria. The registry must be accessible to the public at no charge. It must be open to all prospective registrants and managed by a not-for-profit organization. There must be a mechanism to ensure the validity of the registration data, and the registry should be electronically searchable. An acceptable registry must include at minimum the following information: a unique identifying number, a statement of the intervention (or interventions) and comparison (or comparisons) studied, a statement of the study hypothesis, definitions of the primary and secondary outcome measures, eligibility criteria, key trial dates (registration date, anticipated or actual start date, anticipated or actual date of last follow-up, planned or actual date of closure to data entry, and date trial data considered complete), target number of subjects, funding source, and contact information for the principal investigator. To our knowledge, at present, only www.clinicaltrials.gov, sponsored by the United States National Library of Medicine, meets these requirements; there may be other registries, now or in the future, that meet all these requirements.

Registration is only part of the means to an end; that end is full transparency with respect to performance and reporting of clinical trials. Research sponsors may argue that public registration of clinical trials will result in unnecessary bureaucratic delays and destroy their competitive edge by allowing competitors full access to their research plans. We argue that enhanced public confidence in the research enterprise will compensate for the costs of full disclosure. Patients who volunteer to participate in clinical trials deserve to know that their contribution to improving human health will be available to inform health care decisions. The knowledge made possible by their collective al-

truism must be accessible to everyone. Required trial registration will advance this goal.

Catherine De Angelis, MD, MPH
Editor-in-Chief, *Journal of the American Medical Association*

Jeffrey M. Drazen, MD
Editor-in-Chief, *The New England Journal of Medicine*

Professor Frank A. Frizelle, MBChB, MMedSc, FRACS
Editor, *The New Zealand Medical Journal*

Charlotte Haug, MD, PhD, MSc
Editor-in-Chief, *Norwegian Medical Journal*

John Hoey, MD
Editor, *Canadian Medical Association Journal*

Richard Horton, FRCP
Editor, *The Lancet*

Sheldon Kotzin, MLS
Executive Editor, MEDLINE
National Library of Medicine

Christine Laine, MD, MPH
Senior Deputy Editor, *Annals of Internal Medicine*

Ana Marusic, MD, PhD
Editor, *Croatian Medical Journal*

A. John P.M. Overbeke, MD, PhD
Executive Editor, *Nederlands Tijdschrift voor Geneeskunde (Dutch Journal of Medicine)*

Torben V. Schroeder, MD, DMSc
Editor, *Journal of the Danish Medical Association*

Harold C. Sox, MD
Editor, *Annals of Internal Medicine*

Martin B. Van Der Weyden, MD
Editor, *The Medical Journal of Australia*
